# Usefulness of speckle tracking echocardiography and biomarkers for detecting acute cellular rejection after heart transplantation

**DOI:** 10.1186/s12947-020-00235-w

**Published:** 2021-01-09

**Authors:** Cecilia Beatriz Bittencourt Viana Cruz, Ludhmila A. Hajjar, Fernando Bacal, Marco S. Lofrano-Alves, Márcio S. M. Lima, Maria C. Abduch, Marcelo L. C. Viera, Hsu P. Chiang, Juliana B. C. Salviano, Isabela Bispo Santos da Silva Costa, Julia Tizue Fukushima, Joao C. N. Sbano, Wilson Mathias, Jeane M. Tsutsui

**Affiliations:** 1 Heart Institute (InCor), University of São Paulo Medical School, Av. Dr. Enéas de Carvalho Aguiar, 44, São Paulo, SP 05403-000 Brazil; 2 Fleury Medicine & Health, São Paulo, Brazil

**Keywords:** Heart transplantation, Acute cellular rejection, Speckle tracking echocardiography

## Abstract

**Background:**

Acute cellular rejection (ACR) is a major complication after heart transplantation. Endomyocardial biopsy (EMB) remains the gold standard for its diagnosis, but it has concerning complications. We evaluated the usefulness of speckle tracking echocardiography (STE) and biomarkers for detecting ACR after heart transplantation.

**Methods:**

We prospectively studied 60 transplant patients with normal left and right ventricular systolic function who underwent EMB for surveillance 6 months after transplantation. Sixty age- and sex-matched healthy individuals constituted the control group. Conventional echocardiographic parameters, left ventricular global longitudinal, radial and circumferential strain (LV-GLS, LV-GRS and LV-GCS, respectively), left ventricular systolic twist (LV-twist) and right ventricular free wall longitudinal strain (RV-FWLS) were analyzed just before the procedure. We also measured biomarkers at the same moment.

**Results:**

Among the 60 studied patients, 17 (28%) had severe ACR (grade ≥ 2R), and 43 (72%) had no significant ACR (grade 0 – 1R). The absolute values of LV-GLS, LV-twist and RV-FWLS were lower in transplant patients with ACR degree ≥ 2 R than in those without ACR (12.5% ± 2.9% vs 14.8% ± 2.3%, *p*=0.002; 13.9° ± 4.8° vs 17.1° ± 3.2°, *p*=0.048; 16.6% ± 2.9% vs 21.4%± 3.2%, *p* < 0.001; respectively), while no differences were observed between the LV-GRS or LV-GCS. All of these parameters were lower in the transplant group without ACR than in the nontransplant control group, except for the LV-twist. Cardiac troponin I levels were significantly higher in patients with significant ACR than in patients without significant ACR [0.19 ng/mL (0.09–1.31) vs 0.05 ng/mL (0.01–0.18), *p*=0.007]. The combination of troponin with LV-GLS, RV-FWLS and LV-Twist had an area under curve for the detection of ACR of 0.80 (0.68–0.92), 0.89 (0.81–0.93) and 0.79 (0.66–0.92), respectively.

**Conclusion:**

Heart transplant patients have altered left ventricular dynamics compared with control individuals. The combination of troponin with strain parameters had higher accuracy for the detection of ACR than the isolated variables and this association might select patients with a higher risk for ACR who will benefit from an EMB procedure in the first year after heart transplantation.

## Introduction

Heart transplantation is the treatment of choice for selected patients with end-stage heart failure [1, 2]. Although significant advances in immunosuppressive therapy have been beneficial in decreasing cardiac allograft rejection, graft failure remains one of the major associated complication [3, 4]. For this reason, adequate monitoring of heart transplant patients to diagnose and initiate specific therapy for transplant rejection in a timely manner is important, albeit challenging.

Endomyocardial biopsy (EMB) is the widely accepted gold standard for the diagnosis of acute cellular rejection (ACR) [5]. However, it is invasive and is associated with complications in 0.2 to 5.5% of cases; these complications include tricuspid regurgitation, cardiac perforation and cardiac tamponade [6–9]. Additional limitations of EMB include the subjectivity of the pathological analysis, and significant interobserver variability, which compromises its reliability and reproducibility [10–12].

Therefore, there has been considerable effort exerted to develop noninvasive and accurate methods that can reduce the need for EMB, including biomarker detection, imaging techniques and genetic tests [13, 14]. The most frequently used biomarkers in patients with heart transplantation are troponin and B-type natriuretic peptide (BNP). However, there is considerable heterogeneity among studies about the timing of their use and their predictive value for detecting ACR [15, 16].Two-dimensional speckle-tracking echocardiography (STE) is an echocardiographic modality for the evaluation of myocardial deformation, enabling the determination of multiple parameters involved in cardiac mechanics, and it has emerged as a promising tool for detecting early subclinical cardiac dysfunction in many different scenarios [17, 18]. Previous studies in the heart transplant population demonstrated that, in this setting, the data are not definitive, and further exploration of cardiac mechanics is necessary [19, 20].

In the present study, we aimed to assess the value of STE-derived strain measurements and biomarkers for the noninvasive detection of ACR after heart transplantation. We also evaluated the ventricular dynamics of heart transplant patients in comparison with control individuals using STE.

## Methods

### Patients

From January 2014 to November 2018, we prospectively studied heart transplant patients who underwent EMB in the 6th month after orthotopic heart transplantation for the diagnosis of ACR during routine surveillance. All of the included patients were asymptomatic and did not present with any hemodynamic compromise at the time of enrollment.

The exclusion criteria were as follows: age < 18 years, arrhythmia, left ventricular ejection fraction < 0.55, right ventricular dysfunction of any degree (fractional area change< 35%), vascular graft disease, humoral rejection, two or more previous cellular rejection episodes, chronic kidney disease, Chagas disease reactivation, limited echocardiographic window for STE analysis and an inconclusive EMB analysis.

All patients underwent the same protocol according to the predefined steps. First, enrolled patients underwent a complete echocardiographic analysis for the assessment of left and right ventricular function. For those with a normal systolic function, images were acquired for mechanical analysis by STE, and blood was taken for biomarker tests. The patients then underwent EMB and, according to the results, were divided into the following groups: 1) without significant ACR (grades 0 and 1R) and 2) with significant ACR (grades 2R and 3R).

Individuals without a clinical history of any disease known to interfere with myocardial physiology or structure and who were matched by sex and age with the transplant patients constituted the control group. This group underwent STE for the analysis of ventricular mechanics and was compared to the groups of transplant patients with and without significant ACR. The study protocol was approved by our ethical committee, and all patients gave written informed consent to participate.

### Endomyocardial biopsy

EMB was performed through the internal jugular or femoral vein under radioscopy. A minimum of 3 distinct ventricular myocardial fragments were collected (each consisting of at least 50% myocardium) and sent for anatomopathological analysis. A sample was considered sufficient when at least 3 myocardial fragments were obtained for analysis by optical microscopy after fixation in 10% formalin and staining of the laminae with hematoxylin and eosin. Two experienced cardiac pathologists blinded to the echocardiographic results analyzed all biopsies. The grade of rejection was based upon the recommendations of the International Society for Heart and Lung Transplantation [21]. The results of the EMB were described as grade 0 (without rejection), grade 1R (mild rejection, low grade), grade 2R (moderate rejection, intermediate grade) or grade 3R (severe rejection, high grade) [21]. In our study, grades 2R and 3R were considered as significant ACR.

### Echocardiography

On the day of EMB, just before the procedure, patients underwent echocardiographic examination on a commercially available machine equipped with an MS5 probe (GE Vivid 9, GE Healthcare, Milwaukee, Wisconsin, USA). Image acquisition and assessment were performed according to the recommendations of the American Society of Echocardiography [22]. Left ventricular ejection fraction was obtained by Simpson’s rule throughout apical 4- and 2-chamber views, and the left ventricular mass was calculated using the equation proposed by Devereux et al. [23], indexed by body surface area to derive the left ventricular mass index. Right ventricular systolic function was assessed with the conventional parameters recommended for routine clinical practice: tricuspid annular plane systolic excursion, systolic excursion velocity, and fractional area change, which were obtained with M-mode, pulsed tissue Doppler and two-dimensional echocardiography, respectively. Diastolic function was evaluated based on mitral inflow E/A pattern, E/A ratio, E velocity deceleration time, annular tissue Doppler curves (e’/a’), and E/e’ ratio.

To assess the ventricular mechanics, 3 consecutive cardiac cycles were recorded. Left ventricular short-axis and apical views were acquired using two-dimensional grayscale second-harmonic imaging at a frame rate of 50–80 frames per second. Left ventricular short-axis views at the basal, mid (papillary muscles) and apex levels were acquired to analyze the circumferential (LV-GCS) and radial strain (LV-GRS), while the left ventricular apical 4-, 2- and 3-chamber views were used to assess the left ventricular global longitudinal strain (LV-GLS) and the apical 4-chamber view focused on the right ventricle was used to analyze the right ventricular free wall longitudinal strain (RV-FWLS).

STE analysis was performed offline using dedicated software (EchoPAC, version BT11, GE Healthcare). All echocardiographic measurements were performed by one specialist blinded to the clinical data. End-systole was determined by pulsed-wave Doppler at the time of aortic valve closure. After the ventricular endocardial border was manually traced, the epicardial borders were automatically defined to create regions of interest according to the ventricular segmentation; if necessary, adjustments were made by the operator. In particular, care was taken to not include the myocardial trabeculae and the pericardium. Following this step, the myocardial speckles were automatically tracked by the dedicated software, and, in the case of suboptimal tracking, further manual adjustments were allowed, resulting in strain curves that were exported to a spreadsheet. Global longitudinal strain, circumferential strain, and radial strain were calculated as averages of peak systolic strain values obtained from all segments in the respective views. Accordingly, the LV-GLS was obtained from the mean of 18 segments acquired in the apical 4-, 2-, and 3-chamber views, while circumferential and radial strain were obtained from 12 segments in LV short-axis views at the basal, papillary muscle levels and apex. Left ventricular twist (LV-Twist) is the wringing motion of the heart around its long axis. It was calculated as the net absolute difference between the apical and basal rotations (LV-Twist = ROTapical – ROTbasal). By widely assumed convention, apical rotation has positive values and basal had negative values [24]. The RV-FWLS was obtained by averaging the values of the 3 right ventricular free wall segments: basal, medial, and apical. Care was taken to obtain the best visualization of the right ventricle to enable accurate delineation of its endocardial border. Irregular cardiac cycles or those containing premature ventricular beats were excluded.

The intraobserver reproducibility of the strain measurements was assessed in a subsample of 30 randomly selected patients 3 months after the initial evaluation; the observer was blinded to the previous results. Interobserver variability was assessed in the same subsample by a second blinded experienced echocardiographer.

### Biomarkers

Biomarker analysis was performed before EMB. For this, a 20 mL blood sample was collected from a peripheral veinto determine the plasma levels of cardiac troponin I and BNP.

Cardiac troponin I levels were quantified with a high sensitivity 3-step sandwich immunoassay using direct chemiluminescent technology and consistent amounts of 2 monoclonal antibodies. An auxiliary reagent was included to reduce nonspecific binding using an Advia Centaur TnI-Ultra commercial kit (Siemens Healthcare Diagnostics, Tarrytown, New York, USA). The level of detection was 0.006 ng/mL (levels < 0.006 were reported as 0.005 ng/mL). The normal range of cardiac troponin I was considered < 0.04 ng/mL.

Plasma concentrations of BNP were determined with a 2-step sandwich immunoassay using direct chemiluminescent technology and consistent amounts of 2 monoclonal antibodies using an Advia Centaur commercial kit (Siemens Healthcare, Malvern, Pennsylvania, USA). The level of detection was 2 pg/mL. Levels < 2 were reported as 1 pg/mL.

### Statistical analysis

Categorical variables were compared using Pearson chi-square tests, Fisher exact tests, or likelihood ratio tests. Continuous variables were compared usingthe analysis of variance and Tukey’s test (normal distribution) or the Kruskal-Wallis and Dunn’s tests. The results were expressed as the means with standard deviations or as the medians with interquartile ranges. Linear correlations were tested using the Spearman rank method.

Receiver operating characteristic curves were constructed for variables significantly associated with the presence of ACR degree 2R, and the optimal cutoff values were calculated as the point with the highest sums of sensitivity and specificity. Receiver operating characteristic curves were also constructed for troponin combined with strain parameters significantly associated with the presence of ACR degree 2R.

The interobserver and intraobserver reproducibility of LV-GLS, LV-Twist and RV-FWLSwere assessed using intraclass correlation coefficients and 95% confidence intervals (CIs) in one-way random and two-way mixed models.

All analyses were performed using SPSS version 17 (SPSS Inc., Chicago, Illinois, USA). A *p*-value < 0.05 was considered statistically significant.

## Results

**Fig. 1:**
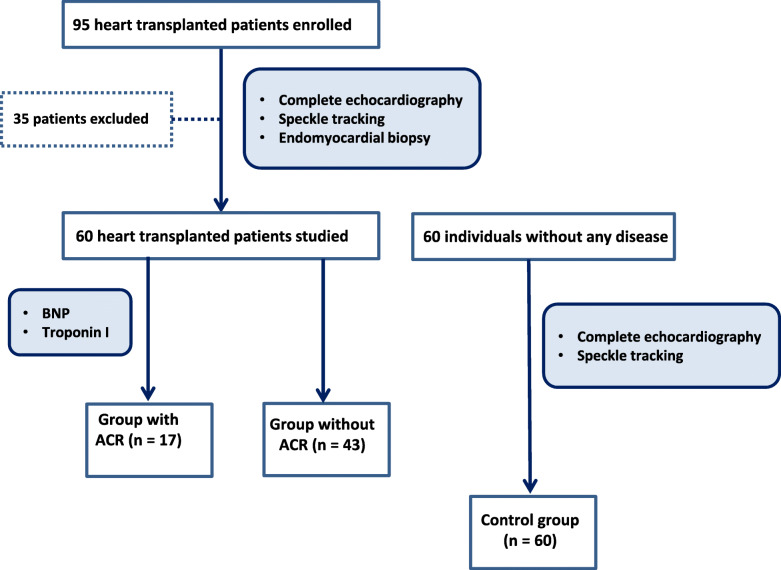
Study flow diagram. EMB = endomyocardial biopsy; ACR = acute cellular rejection

Ninety-five patients were initially enrolled in the study. Among these patients, 35 were excluded because of the following characteristics: 2 due to cardiac arrhythmia, 4 due to left ventricular systolic dysfunction, 4 due to right ventricular systolic dysfunction, 14 due to two or more previous ACR episodes, 4 due to a limited echocardiographic acoustic window for STE analysis, 3 due to inconclusive results of EMB, 2 due to humoral rejection and 2 due to Chagas disease recurrence. A total of 60 heart transplant patients and 60 control individuals constituted the final study population (Fig. 1).

We analyzed a total of 60 EMBs of heart transplant patients. All biopsies were performed at 6 months after heart transplantation. Among these 60 EMBs, 43 (72%) did not show significant ACR (grade 0 or 1R), while 17(28%) showed significant ACR (grade 2R or 3R).

The baseline characteristics of the heart transplant patients included in this study are described in Table 1. The clinical characteristics of the groups with and without significant ACR detected by EMB, as well as those of the control group individuals, are described in Table 2.

**Table 1:** Baseline characteristics of the transplant patients

Characteristic	Heart transplant patients
	*n*= 60
Male sex	34 (56.7%)
Age (years)	42.1± 11.5
Weight (kg)	61.3 ± 13.4
Height (cm)	162.5 ± 10.3
Body surface area (m^2^)	1.7 ± 0.2
Primary disease	
Chagasic cardiomyopathy	22 (36.7%)
Idiopathic dilated cardiomyopathy	21 (35.0%)
Ischemic cardiomyopathy	7 (11.7%)
Valvular cardiomyopathy	5 (8.3%)
Hypertensive cardiomyopathy	4 (6.7%)
CTRCD	1 (1.6%)
Hypertension	12 (20.0%)
Diabetes	12 (20.0%)
Smoking	17 (28.3%)
Time since transplantation (months)	6
Medication	
Corticosteroid	63 (95.5%)
Mycophenolate mofetil	62 (93.9%)
Tacrolimus	45 (68.2%)
Cyclosporine	21 (31.8%)
Sirolimus	4 (6.1%)
Azathioprine	4 (6.1%)
Statin	61 (92.4%)
CCB	56 (84.5%)
Betablocker	11 (16.7%)
ACEI/ARB	11 (16.7%)

The values are presented as the mean (standard deviation) or number (percentage)

*CTRCD* Cancer therapeutic-related cardiac dysfunction; *ACEI* angiotensin converting enzyme inhibitor; *ARB* aldosterone receptor blocker; *CCB* calcium channel blocker

**Table 2:** Characteristics of the control group and groups with and without significant ACR

Variable	Control group	Group without	Group with	*p* value
	(*n*=60)	significant ACR (*n*=43)	significant ACR (*n*=17)	
Age (years)	43±9	44±11	48±13	0.20 ^c^
Male sex	34 (56.7%)	24 (55.8%)	10 (58.8%)	0.139^b^
Weight (kg)	72 (63–80)	70 (58–81)	71 (61–87)	0.684ª
Height (cm)	1.69±0.09	1.62 ± 0.09^#^	1.59 ± 0.13^#^	< 0.001^c^
BMI (kg/m^2^)	24.7 (22.9–27.1)	26.0 (22.7–31.6)	26.1 (24.0–34.7)	0.152ª
SBP (mmHg)	123 ± 13	126 ± 19	122 ± 13	0.672^c^
DBP (mmHg)	76 ± 10	76 ± 10	72 ± 8	0.463^c^
PAP (mmHg)	–	30 ± 10	34 ± 11	0.465^d^
Hypertension	0 (0%)	7 (16.3%)	5 (29.4%)	< 0.001^b^
Diabetes mellitus	0 (0%)	7 (16.3%)	5 (29.4%)	< 0.001^b^
Smoking	0 (0%)	14 (32.6%)	3 (17.6%)	< 0.001^b^
Cr (mg/dL)	0.80 (0.5–1.0)	1.19 (0.9–1.34)	1.1 (0.95–1.4)	0.930 ª
BSA (m^2^)	1.60 (1.55–1.68)	1.78 (1.58–1.93)^#^	1.75 (1.60–2.03)^#^	< 0.001ª

The values are presented as the mean (standard deviation), median (interquartile range) or number (percentage). *BMI* body mass index; *Cr* creatinine; *BSA* body surface area; *SBP* Systolic blood pressure; *DBP* diastolic blood pressure; *PAP* pulmonary artery pressure; *ACR* acute cellular rejection

^a^Kruskal-Wallis test; ^b^Pearson chi-square; ^c^ANOVA

#*p*< 0.05 (vs. the control group)

### Biomarkers

Cardiac troponin I levels as assessed by the ultrasensitive assay were significantly higher in patients with significant ACR than in patients without significant ACR [0.19 ng/mL (0.09–1.31) vs 0.05 ng/mL (0.01–0.18), *p*=0.007]. No significant difference in BNP levels was found between patients with significant ACR and patients without significant ACR [264.5 pg/mL (160 to 976) vs 248 pg/mL (90 to 528), *p*=0.435, respectively].

### Speckle tracking echocardiography

The variables obtained by conventional echocardiography are shown in Table 3. There were no differences between the group with significant ACR and the group without significant ACR regarding these parameters. However, heart transplant patients had a greater septum and inferolateral wall thickness, a larger left atrium diameter, a higher E/E’, and higher relative thickness and left ventricular mass index values than control individuals. In addition, the heart transplant patients had a lower right ventricular fractional area change, systolic velocity of the tricuspid annulus, E’ velocity, A’ velocity and tricuspid annular plane systolic excursion than control individuals.

**Table 3:** Echocardiographic parameters and their relationships with significant acute cellular rejection

Variable	Control	Group without ACR	Group with ACR	p value
	group			
Left atrium (mm)	34 (31–36)	41 (38–46)#	43 (35–49)#	< 0.001ª
LA Volume (mm^3^/cm^2^)	25.02 ± 2.97	33.31 ± 4.96#	33.78 ± 3.06#	< 0.001^b^
Septum (mm)	8 (8–9)	11 (10–13)#	12 (10–13)#	< 0.001ª
Inferolateral wall (mm)	8 (8–9)	11 (10–11)#	11 (9–13)#	< 0.001ª
Relative thickness	0.38 (0.35–0.41)	0.49 (0.44–0.53)#	0.47 (0.40–0.55)#	< 0.001ª
LVMI (g/m^2^)	71 (65–83)	102 (86–124)#	109 (80–141)#	< 0.001ª
LVEF (%)	65 (62–68)	64 (62–67)	65 (63–67)	0.739ª
E velocity (m/s)	0.84 (0.69–0.93)	0.65 (0.60–0.76) #	0.66 (0.54–0.84) #	< 0.001ª
DT (ms)	189 (168–208)	187 (144–214)	190 (153–207)	0.787ª
A velocity (m/s)	0.50 (0.42–0.58)	0.41 (0.32–0.50) #	0.42 (0.38–0.68) #	0.001ª
E/A	1.60 (1.38–1.93)	1.64 (1.29–2.00)	1.46 (1.29–1.75)	0.543ª
S′ velocity (m/s)	0.08 (0.07–0.09)	0.07 (0.06–0.08)#	0.07 (0.05–0.08)	0.010ª
E’ velocity (m/s)	0.11 (0.09–0.12)	0.06 (0.05–0.08)#	0.07 (0.05–0.07)#	< 0.001ª
A’ velocity (m/s)	0.08 (0.07–0.10)	0.06 (0.05–0.08)#	0.08 (0.06–0.09)#	< 0.001ª
E/E’	7.92 (6.63–8.78)	10.14 (7.99–12.96)#	10 (7.93–14.21)#	< 0.001ª
FAC (%)	43 (40–48)	39 (36–45) #	39 (37–42) #	0.001ª
TAPSE (mm)	24 (20–26)	17 (15–18)#	16 (15–17)#	< 0.001ª
S′ velocity (m/s) of tricuspid annulus	13 (12–15)	12 (11–14)	11 (11–12)#	< 0.001ª

The values are presented as the mean (standard deviation) or median (interquartile range)

^a^Kruskal-Wallis test;#*p*< 0.05 (vs. the control group). *ACR* acute cellular rejection; *LA* left atrium; *DT* deceleration time; *LVEF* left ventricular ejection fraction; *FAC* fractional area change; *lat* lateral; *LVMI* left ventricular mass index; *TAPSE* tricuspid annular plane systolic excursion

**Fig. 2:**
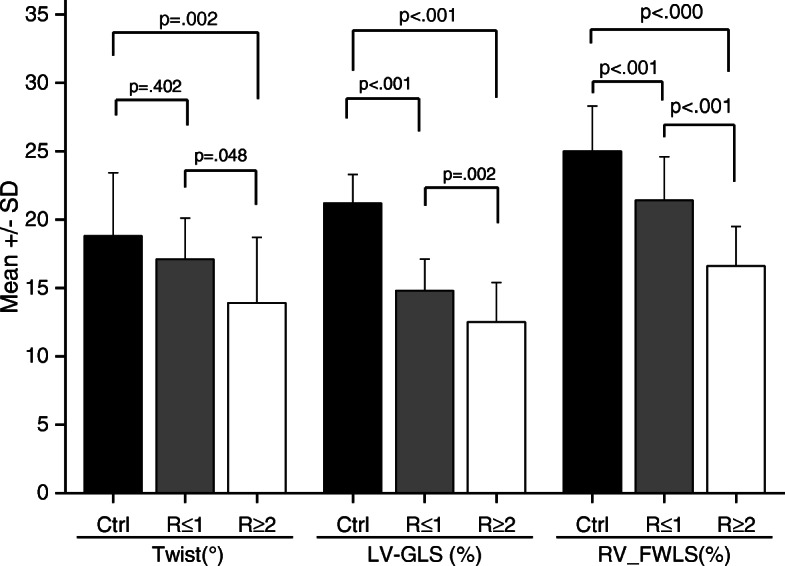
Twist, left ventricular global longitudinal strain and right ventricular free wall longitudinal strain in the control group and in two groups of heart transplant patients, those with and those without significant acute cellular rejection (R ≥2 vs. R ≤1) ANOVA: F-test. *Significative to 5% (Tukey test for all pairwise comparisons); 1: Ctrl = Control (Cardiac untransplanted patients); 2: Heart transplant patients without rejection (R ≤1); 3: Heart transplant patients with rejection (R ≥2). SD: Standard Deviation; LV-GLS: Left Ventricular Global Longitudinal Strain; RV-FWLS, Right Ventricular Free Wall Longitudinal Strain

The LV-GLS, LV-GCS, LV-GRS, RV-FWLS and LV-twist values are shown in Table 4. The absolute values of these variables were significantly lower in the heart transplant patients without rejection than in the control individuals, except for the LV-twist. In the group with significant ACR, the LV-GLS, LV-twist and RV-FWLS were significantly lower (in absolute values) than that in the groups without significant ACR (12.5% ± 2.9% vs 14.8% ± 2.3%, *p* = 0.002;13.9° ± 4.8° vs 17.1° ± 3.0°, *p* = 0.048;16.6% ± 2.9%vs21.4%± 3.2%, *p*< 0.001; respectively), as shown in Fig. 2.

**Table 4:** LV-twist, LV-GLS, LV-GCS, LV-GRS and RV-FWLS values and their relationships with cardiac rejection in univariate analysis

Variable	Control	Without	With	*P*^*†*^	*P*^*‡*^	P^*^	*Overall*
	group	ACR	ACR				
	(*n*=60)	(*n*=43)	(*n*=17)				*P value*^*§*^
LV-twist (^o^)	18.8 ± 4.63	17.1 ± 3.02	13.9 ± 4.79	0.402	0.002	0.048	0.001
LV-GLS (%) ^AV^	21.2 ± 2.1	14.8 ± 2.3	12.5 ± 2.9	< 0.001	< 0.001	0.002	< 0.001
LV-GCS (%)^AV^	21.3 ± 3.0	16.2 ± 4.3	15.2 ± 2.8	< 0.001	< 0.001	0.574	< 0.001
LV-GRS (%)	41.2 ± 12.9	28.6 ± 9.9	28.3 ± 8.8	< 0.000	< 0.001	0.996	< 0.001
RV-FWLS (%)^AV^	25.0 ± 3.3	21.4 ± 3.2	16.6 ± 2.9	< 0.000	< 0.001	< 0.001	< 0.001

*AV* absolute value; *ACR* acute cellular rejection

Data are expressed as the mean ± SD

† Control group vs group without significant ACR

‡Control group vs group with significant ACR

*Group without ACR vs group with significant ACR

§ Analysis of variance global P value between groups

**Fig. 3:**
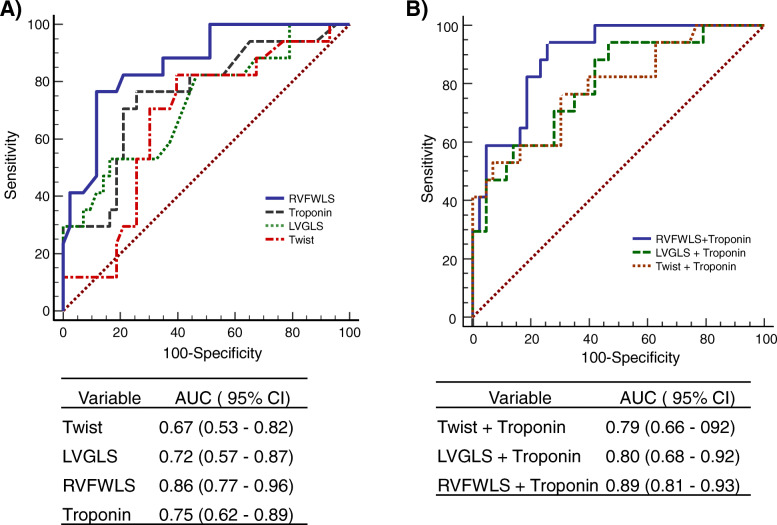
**a** Receiver operating characteristic curve demonstrating troponin, the right ventricular free wall longitudinal strain**,** left ventricular global longitudinal strain and left ventricular twist values obtained by speckle-tracking echocardiography for the diagnosis of acute cellular rejection in heart transplant patients. **b** Receiver operating characteristic curve demonstrating troponin combined with right ventricular free wall longitudinal strain**,** left ventricular global longitudinal strain and with left ventricular twist values obtained by speckle-tracking echocardiography for the diagnosis of acute cellular rejection in heart transplant patients. AUC = area under curve; CI = confidence interval; LV GLS, left ventricular global longitudinal strain; RV FWLS, right ventricular free wall longitudinal strain

When we combined the strain parameters assessed as significant to detect ACR with troponin, we found that it increased the accuracy compared with the variables used individually. We report it in the model described below, based on the area under the curve (AUC) (Fig. 3).

The intraclass correlation coefficient of LV-GLS was 0.92 (95% CI = 0.85–0.96) for interobserver variability. For intraobserver variability, the intraclass correlation coefficient of the LV-GLS was 0.93 (95% CI = 0.86–0.97). The intraclass correlation coefficients of the RV-FWLS were 0.90 (95% CI = 0.80–0.95) and 0.92 (95% CI = 0.84–0.96) for inter- and intraobserver variability, respectively. For the LV-Twist, the inter and intraobserver variability were 0.79 (95% CI = 0.35–0.94) and 0.78 (95% CI = 0.33–0.94), respectively.

## Discussion

The main results of our work are that patients with ACR had significantly lower values of LV-GLS, RV-FWLS and LV-Twist and higher level of troponin I than patients without significant ACR. In addition, the combination of troponin and these parameters may improve the diagnostic value of strain in heart transplant patients with preserved left and right ventricular systolic functions. The combination of RV-FWLS and troponin presented a higher accuracy for the detection of ACR degree ≥2R. The AUC of the combination of troponin (> 0.05 ng/mL) with RV-FWLS (with a cut-off value < 18%) was 0.89(95%CI = 0.81–0.93). Thus, it may be considered for use as a screening tool for the detection of ACR and could reduce the number of EMBs after heart transplantation.

ACR is a significant and frequent complication of heart transplantation. In the first year, it is the most common cause of mortality. Currently, EMB is the clinical gold standard in screening for graft rejection after heart transplantation and is actually the only tool for the diagnosis and classification of allograft rejection [5]. Considerable efforts have been made to improve the consistency, reliability and reproducibility of the histopathological evaluation of EMB. However, several issues make EMB assessment more difficult and less reproducible than it should be. Critical issues include the subjective and challenging pathological interpretation of EMBs and the risks associated with the procedure [25]. Considering these limitations, noninvasive techniques to detect cardiac rejection have been evaluated [14, 26].

To the best of our knowledge, this is the first study to analyze all strain parameters using STE (LV-GLS, LV-GRS, LV-GCS, LV-Twist and RV-FWLS) in the same population of heart transplanted patients with normal right and left ventricular systolic function, and its combination with biomarkers, to diagnosis clinically significant ACR. Extra care was given in our study to restrict patient selection to a fixed period of time (6 months post-heart transplantation) to minimize the possible influence of the time since heart transplantation on ventricular strain, as recommended by the current guidelines [27]. Additionally, this fixed period of selection minimized the possible bias of pre transplantation ischemic injury, which can manifest up to the sixth month post heart transplantation. The first 6 months is a period of adaptation, during which many patients can still present some degree of right ventricular systolic dysfunction.

Previous studies have shown that myocardial strain has a higher sensitivity than conventional echocardiography, and therefore, may be an important tool to detect early subclinical cardiac dysfunction [28]. Although myocardial strain imaging has been reported to have potential for the detection of graft dysfunction in the early stage, its diagnostic value has not been widely recognized yet [19, 20]. Similarly, as reported in a recent meta-analysis, our study showed that the LV-GLS was lower in transplant patients with significant ACR compared with patients without significant ACR [29]. This may occur secondary to myocardial deformation, and it may be impaired due to inflammatory cellular infiltration and myocardial edema, and can be reflected by myocardial strain parameters. However, the diagnostic value of other strain parameters by 2D STE on ACR detection is still controversial.

Right ventricular strain analysis has been poorly described to date in this scenario. Mingo-Santos et al. demonstrated a predictive role of STE parameters in the diagnosis of ACR (RV-FWLS and LV-GLS with threshold values of < 17 and < 15.5%, respectively) [30]. That study classified biopsies into 3 groups (0, 1R, and ≥ 2R). However, our study divided the biopsy results into two groups according to the grade of rejection: biopsies without significant rejection (0 and 1R) and biopsies with significant rejection (≥ 2R). This division was based upon the clinical meaning of the rejection grade, since cases with grades of 0 or 1R do not require an immediate intervention via adjustment of immunosuppressive medications, whereas this adjustment is necessary in patients presenting with 2R and 3R rejection. In agreement with the study from Mingo-Santos et al. [30],our study confirms the reduction in left ventricular and right ventricular STE parameters during ACR ≥ 2. Our cutoff value was slightly higher than that reported by Mingo-Santos et al. [30]. We speculate that it might be due to the use of different echocardiographic equipment or even the characteristics of the studied populations. Unfortunately, the investigators did not analyze LV-Twist and troponin.

As we showed in our results, the LV-Twist values were significantly lower in the group with significant ACR than in the group without significant ACR (13.9° ± 4.79° vs 17.1° ± 3.02°, *p* < 0.048). In parallel to our results, a unique previous study that applied STE derived LV-Twist measurements to detect rejection in heart transplanted patients demonstrated that the LV-Twist decreased more in the group with ACR than in the group without ACR (9.6°±2.7° vs 12.2° ±2.3°), *p* < 0.0001, [31]. We postulated that twist preceded the deterioration in left ventricular ejection fraction, suggesting early myocardial involvement in cardiac rejection. With the advances of technology that have made this technique more available and increasingly feasible, this parameter of cardiac mechanics has been increasingly studied in other pathological situations, and can be applied in this type of patient [32, 33].

As acute rejection promotes cardiomyocyte necrosis and results in compromised cardiac mechanics, troponin and BNP have been evaluated as potential diagnostic tools for ACR [34, 35]. There have been controversial results on these biomarkers in the field of heart transplantation [15, 36]. Our study used an ultrasensitive assay for cardiac troponin I that detects 10 to 100 times lower levels than standard assays. Troponin was measured before the biopsy, so this procedure did not interfere with its serum levels. Troponin I levels were significantly elevated in patients with significant ACR. Serum BNP levels were not different between the groups; this finding can be explained by the suggestion of some studies that BNP remains altered in most patients for up to 1 year after heart transplantation [37, 38]. In accordance with Bader et al. [39], we also observed that BNP levels did not predict rejection after heart transplantation, and we suggest that BNP is not clinically useful for the detection of ACR.

In accordance with previous reports in the literature [40], we confirmed in our population that heart transplant patients have a characteristic cardiac geometric remodeling, featured by a greater septum and inferolateral wall thickness, a larger left atrium diameter, and greater left ventricular mass index values than matched non transplanted controls. Heart transplant patients also showed lower values for conventional right ventricular systolic function parameters, such as fractional area change, tricuspid annulus systolic velocity and tricuspid annular plane systolic excursion. Moreover, regarding left ventricular diastolic function, heart transplant patients had lower tissue Doppler velocities and higher E/e´ ratios, suggesting impaired relaxation and increased left ventricular filling pressures.

This study demonstrated that heart transplant patients without rejection present unique ventricular dynamics, characterized by lower LV-GLS, LV-GCS, LV-GRS and RV-FWLS, in comparison with control individuals. We have confirmed the data recently published by Ingvarsson et al. [40], which showed that echocardiographic measurements from 124 heart transplant patients were different from the reference values except for LV-GCS. Unfortunately, the investigators did not analyze LV-Twist. In our study, we used a non-transplanted control group matched by age and sex to confirm these results. Multiple mechanisms may explain the different echocardiographic findings in heart transplant patients. Their pathophysiology involves the consequences of surgical trauma, such as ischemic injury and the release of inflammatory mediators, in addition to previous pulmonary hypertension compromising right ventricular dynamics and the risks associated with rejection, cardiac biopsies and immunosuppressive medications.

Our data failed to find any association between diastolic markers and rejection. The results found in the literature are highly conflicting and could not be reproduced by our data. This can be explained by a limitation of diastolic dysfunction parameters due to their dependence on heart rate (which is generally elevated in transplanted patients, with a fusion of E and A waves), loading conditions and donor age [41–45].

### Limitations

The limitations of this study should be addressed. First, this was a single-center study with a small number of patients and a limited number of rejection episodes graded equal to or above 2R (17 out of 60 samples). Despite its extensive validation, STE is still an evolving technique, and improvements such as better tracking accuracy are still needed. Additionally, STE accuracy is highly dependent on image quality. Suboptimal resolution can produce a negative impact on the final results. Nevertheless, despite these limitations, we were able to successfully perform speckle-tracking analysis of both left and right ventricular longitudinal strain in 95% of the patients. The reproducibility of the parameters was good and was similar to that reported in other studies. Finally, our results must be independently validated in a prospective external cohort, preferably in multicenter studies, before they can be used in clinical practice.

## Conclusions

Heart transplant patients have altered left ventricular dynamics compared with control individuals. The association of strain parameters derived from STE, particularly RV-FWLS, with troponin seems to be able to detect ACR with higher accuracy than these variables used individually. These parameters might be considered a screening method that can be added to the management of heart transplant patients to safely reduce the number of unnecessary EMB.

### Perspectives

With the increase in heart transplantation, new tools for detecting acute cellular rejection are highly desirable. Our study findings show that the analysis of strain parameters with troponin levels might be useful to noninvasively detect patients who are at high risk of cellular rejection.

## Data Availability

All data generated or analysed during this study are included in this published article. If you have questions or additional information, the datasets used and/or analysed during the current study are available from the corresponding author upon reasonable request.
