# Correction to: Usefulness of speckle tracking echocardiography and biomarkers for detecting acute cellular rejection after heart transplantation

**DOI:** 10.1186/s12947-021-00241-6

**Published:** 2021-01-28

**Authors:** Cecilia Beatriz Bittencourt Viana Cruz, Ludhmila A. Hajjar, Fernando Bacal, Marco S. Lofrano-Alves, Márcio S. M. Lima, Maria C. Abduch, Marcelo Luiz Campos Vieira, Hsu P. Chiang, Juliana B. C. Salviano, Isabela Bispo Santos da Silva Costa, Julia Tizue Fukushima, Joao C. N. Sbano, Wilson Mathias, Jeane M. Tsutsui

**Affiliations:** 1 Heart Institute (InCor), University of São Paulo Medical School, Av. Dr. Enéas, de Carvalho Aguiar, 44, São Paulo, SP 05403-000 Brazil; 2 Fleury Medicine & Health, São Paulo, Brazil

**Correction to: Cardiovasc Ultrasound (2021) 19:6**

**https://doi.org/10.1186/s12947-020-00235-w**

Following publication of the original article [1], the authors identified an error in the author name of Marcelo L. C. Vieira.

The incorrect author name is:

Marcelo L. C. Viera.

The correct author name is:

Marcelo L. C. Vieira.

The original article [1] has been corrected.
